# Process-Structure Coupled Simulation of Additive Manufactured Components

**DOI:** 10.3390/polym15040949

**Published:** 2023-02-14

**Authors:** Fabian Ferrano, Tizian Wachter, Miranda Fateri, Michael Schmiedt

**Affiliations:** 1 Faculty Mechanical Engineering & Materials Science, Aalen University, 73430 Aalen, Germany; 2 Technology Center Lightweight Construction, Faculty Mechanical Engineering & Materials Science, Aalen University, 73430 Aalen, Germany

**Keywords:** additive manufacturing, 3D printing, fused layering manufacturing, digital manufacturing, FEM simulation, manufacturing simulation

## Abstract

In this work, the influence of extrusion infill angles on the mechanical properties of 3D printed (Fused Filament Fabrication, FFF) test specimens are investigated, considering the real geometry of the components. Therefore, various polylactide (PLA) specimens with different infill angles are manufactured, scanned by Computed Tomography (CT) and further investigated by mechanical testing using an optical measuring system. This allows the directional dependence and the elastoplastic behavior of the material to be demonstrated. It was found that the real geometry behavior differs significantly from the model. In addition to the tests Finite Element Method (FEM) simulations of the scanned components are carried out in order to provide a prediction of the mechanical properties of FFF-printed parts for component manufacturers. The conducted simulations have shown that the geometric deviation leads to an increase in stiffness, a higher ultimate tensile strength and strain at failure. The main objective of this work is to evaluate the stiffness and strength of FFF-printed components using FEM with an economically justifiable testing effort. This includes not only the evaluation of the directional dependence, considering the real geometry of the components, but also the evaluation of a suitable strength criterion. The criterion of maximum principal strain has proved to be suitable.

## 1. Introduction

Additive manufacturing (AM) has become an increasingly popular process in recent years [1], not only in industry but also in the private sector. AM can be used to produce a wide range of products, from prototypes to functional components [2]. As such, the technology can be used for almost any field of application as AM does not require tools or specific molds [3].

One of the well-known AM extrusion based processes is Fused Filament Fabrication (FFF), also known as Fused Deposition Modeling (FDM), which is classified as Material Extrusion (MEX) technology according to ASTM 52,900 [4,5]. FDM is the registered/protected trade name for the FFF process. An FFF machine is equipped with an extrusion head (nozzle) and a build platform. The build material for FFF is a pre-fabricated plastic filament, typically 1.75 or 2.85 mm in diameter. In the FFF process, the filament is continuously fed into a nozzle which heats up the feed material above its softening temperature using an electric heater. The softened material is then deposited atop the build platform. The x, y, z profiles of the geometry can be generated by either moving the nozzle or the build platform, or a combination of both. The input information to guide the path of a nozzle and/or build platform is digitally defined by Computer-Aided-Design (CAD) software is used to generate STL files [3].

The FFF process is a relatively simple, easy to use and affordable (due to the lack of a laser or scanner unit when being compared to other AM techniques), which initially made it suitable for the production of non-functional prototypes and personal items [6]. More recently, FFF is entering the industrial world with the development of high-strength filaments and the exploration of new application areas for FFF such as medicine [7].

Therefore, it is crucial to investigate the mechanical properties of the FFF printed components in detail [8]. Compared to conventional manufacturing processes such as injection molding, the FFF process does not require complete melting of the material and the layered structure results in a different mechanical behavior. Furthermore, the orientation of the extruded strands influences the mechanical behavior of the printed component [9], resulting in an anisotropic material behavior of the printed products. Therefore, many researchers have investigated the effect of process parameters on the mechanical properties of the FFF products. Among the published studies, Dudescu et al. experimentally investigated the effect of different infill patterns as well as raster orientation on the tensile properties of 3D printed Acrylonitrile Butadiene Styrene (ABS) specimen. As reported by the authors, the apparent Young’s modulus increases as the percentage of infill increases. The increase per percentage point of infill changes non-linearly, the higher the infill rates the smaller the increase. It is also reported that the effect of void geometry on the local stresses and strains affect the macroscale mechanical behavior of the material [10].

Furthermore, anisotropic mechanical properties of ABS parts fabricated by FFF using different raster orientations are reported by Ziemian et al. In this study, tensile, compression, flexure (3-point bending), impact, and tensile-tension fatigue analyses are performed experimentally on 3D printed specimens. As reported by the authors, the presence of air gaps and the amount of air voids between the raster or fibers influence the strength and effective moduli in all tests [11].

To simulate the FFF process, Xia et al. used a finite volume/front tracking method originally introduced by Unverdi and Tryggvason [12] in 1992 for fully resolved simulations of the deposition of a filament on a substrate. The method is extended to FFF by implicitly handling realistic material parameters, in which the viscosity of the melt is assumed to be a function of temperature and shear rate, and a volume source is incorporated to model the nozzle [13]. In terms of optimizing the process parameters of FFF, Zhou et al. used an FEM simulation approach based on voxelization modelling [14]. In this published research, the influence of key process parameters of FFF on the temperature field, including processing speed, mold chamber temperature and nozzle temperature, is reported.

For the Polylactide (PLA) material, Dobos et al. investigated the effect of infill density and pattern on the specific load capacity of FFF manufactured parts. It is reported that the load capacity efficiency increases at a filling density of 40%, regardless of the filling pattern [15]. Further, Hanon et al. investigated the tensile strength of FFF printed PLA samples using six raster directions, three build orientations and two filling ratios. The optimum print setting was reported to be a crossed 45°/−45° raster direction, X orientation and 100% fill ratio [16]. Regarding part distortion in the FFF process, different studies have focused on simulation and analytical analysis of warpage of 3D printed products [17,18,19].

Overall, the main investigations reported so far have focused on analyzing the initial CAD file geometries for simulations and comparing the simulation data with the collected experimental data. The aim of the present investigation is to determine the effect of different infill strand orientations on the mechanical properties of FFF additive manufactured parts using real geometries.

For this purpose, several PL components with different strand orientations are 3D printed, geometrically measured using CT scans and further examined using quasi static tensile tests. Another objective is to investigate the corresponding mechanical behavior in terms of directionality, elastoplastic material behavior and the difference between real and CAD geometry. By determining the respective material properties and geometries, FEM simulations are carried out to predict the deformation behavior and failure of the tensile test specimen produced by the FFF process. This in turn results in a material model that can be applied to any component geometry and that can describe both the anisotropy due to different infill strand orientations and the deformation behavior in the simulation with sufficient accuracy.

## 2. Materials

In this study, Polylactide is used to produce the tensile specimens. This semi-crystalline bioplastic is mainly produced from renewable resources such as corn starch and lactic acid. However, biodegradation of the material is only possible under certain composting conditions in industrial plants [20]. Among the commonly developed plastics for FFF, PLA offered various applications due to its relatively high UV resistance and severe flammability. In addition, the relatively low moisture absorption tendency of PLA, makes it easy to store. Likewise, the bioplastic exhibits little to no warpage, when being compared to many other common FFF plastics such as ABS.

## 3. Test Sample Preparation

**Figure 1:**
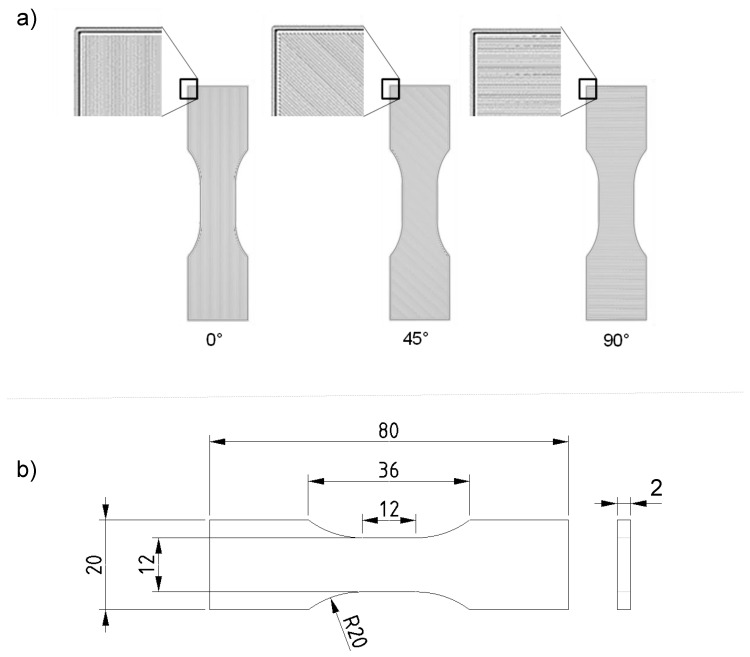
(**a**) Infill angle orientations and (**b**) dimensions of the FFF manufactured tensile test specimens [21].

For the investigation of different infill strand orientations by mechanical testing, five different specimen types are considered, produced by the FFF process. Of these, three test specimens have a unidirectional structure and two have an alternating structure. The unidirectional specimens exhibit orientations of 0°, 45° and 90° to the tensile loading direction (see Figure 1). In principle, the pure layer direction is to be investigated in order to evaluate the properties of these main directions. This should be the basis for evaluating the stiffness and strength in process-structure-coupled simulations, which could form the basis for future research.

The alternating tensile specimens feature a combination of the main orientations. The first variant is a combination of 0° and 90° orientations. Here, the first layer has an orientation of 0° and the second layer 90°, as commonly used in FFF printing. Usually, the infill orientation needs to be rotated by 90 degrees alternately between the layers. This structure is continued until the maximum thickness of the printed object is reached. The second variant follows the same principle. Here, the first layer has an orientation of 45° and the second 135°.

The tensile specimens have a thickness of 2 mm and are printed from PLA with a layer thickness of 0.1 mm. The geometry of the tensile specimens is described in [21] and shown in Figure 1. The advantage over a specimen according to DIN EN ISO 527 1A [22] is that the strain can be measured optically more easily, since the measuring range of the specimen is wider and shorter. The test specimens with the different infill strand orientations are all printed with the same manufacturing parameters to ensure the comparability of the individual tensile specimens. Similarly, each tensile specimen is printed individually with the largest contact area of the tensile specimen centered on the printing platform. The central positioning favors reproducibility as a more constant temperature of the printing bed can be established in this position [23]. For each position, 15 parts are produced and then tested for mechanical properties.

The specimens are printed with the Anycubic i3 Mega 3D printer. A personal printer, as defined by Gebhardt et al. [3], is used in this study since not only a material commonly used for the FFF process is used, but also a widely used printer with the aim of producing technical components that are exposed to high stresses. The quality of the tensile test specimens and the associated low scatter of the mechanical material properties are ensured by the following steps: The 3D printer is sealed in a closed environment in order to ensure constant manufacturing temperature and humidity conditions during printing. In addition, the filament is used immediately after removal from a package in which the material is in a “dry as molded” state to ensure low water absorption of the PLA material. The moisture content is measured after production according to DIN EN ISO 15512:2019 [24] (calcium hydride method). Five measurements are carried out indicating an average water content of 0.14% per sample.

The process parameters for the 3D printed specimens are listed in Table 1. A so-called temperature tower was used to determine the extrusion temperature. This provides specific characteristics that can be used to analyze the optimum extrusion temperature. The evaluation of the temperature tower for the PLA filament resulted in an extrusion temperature of 220 °C. According to the technical data sheet [25], a temperature of 210–230 °C is recommended. The selected extrusion temperature is thus below the maximum recommended temperature, which means that material decomposition can be avoided. The melting temperature is increased by +5 °C for the first printing layer to ensure better adhesion to the printing bed. The temperature of the printing plate should always reflect the glass transition temperature $T_{G}$. The state of the filament in this temperature range is both soft and malleable. This ensures sufficient adhesion of the print and prevents thermal distortion.

A criterion for selecting the layer thickness is that the surface quality and tensile strength can be increased by reducing the layer thickness. Furthermore, a lower layer thickness results in a better homogeneity of the individual layers, which leads to a more effective stress transfer in the component. This behavior has been demonstrated in the study by P. Subham et al. [26]. For this reason, a layer thickness of 0.1 mm was chosen for the manufacture of the tensile test specimens. In addition, the thickness should be less than the diameter of the nozzle in order to allow a certain contact pressure between the individual layers [27].

## 4. Material Testing and Reverse Engineering

In order to make a realistic prediction with the FEM software Abaqus^®^, the tensile specimens with different strand orientations are scanned using Computer Tomography (CT scan). CT measurements are deliberately used to achieve maximum accuracy. Both handheld and desktop scanners do not provide sufficiently accurate measurement results. All optically based measurements are therefore verified with tactile measurements.

**Figure 2:**
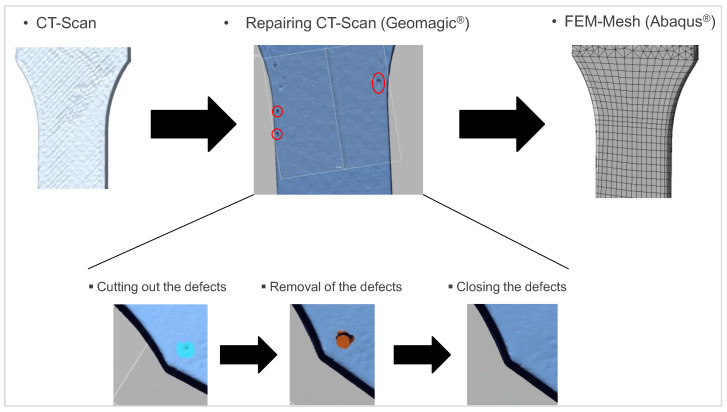
Repair of defects for preparing a closed solid model for FEM simulation.

The aim of reverse engineering is to reconstruct the actual geometries of the specimens as the input data for the FEM simulation. For this purpose, the surface reconstructions are reverse engineered into a solid model using the Geomagic Design X software. The software is also used to process defects resulting from the CT scan since the closing of flaws and holes (defects) in particular is essential for the generation of the solid model. Defects are particularly visible on large, flat surfaces. It is therefore important to ensure that the surface is completely closed. The defect areas are cut out and then closed with a curvature factor. Figure 2 shows different steps to repair the geometric defects of the scanned part. In the left figure, the manufacturing direction of 45° can be seen, but not the defect that needs to be repaired. These defects can be identified in the software first. It is then possible to close the holes manually by marking them, determining the neighboring surfaces using triangulation and defining the outer and inner surface areas using the normal vectors of the individual triangular surfaces. The defects are then closed using multiple triangle elements. Once all the holes are closed, the software checks whether the surface model can be converted into a solid model and then transferred to a CAD program using common data formats. The closed solid model (without holes) is prepared for the subsequent FEM simulation. A model prepared this way is shown in Figure 2 on the right. In addition, the repaired, real geometry can then be compared with the CAD geometry and the differences visualized.

**Figure 3:**
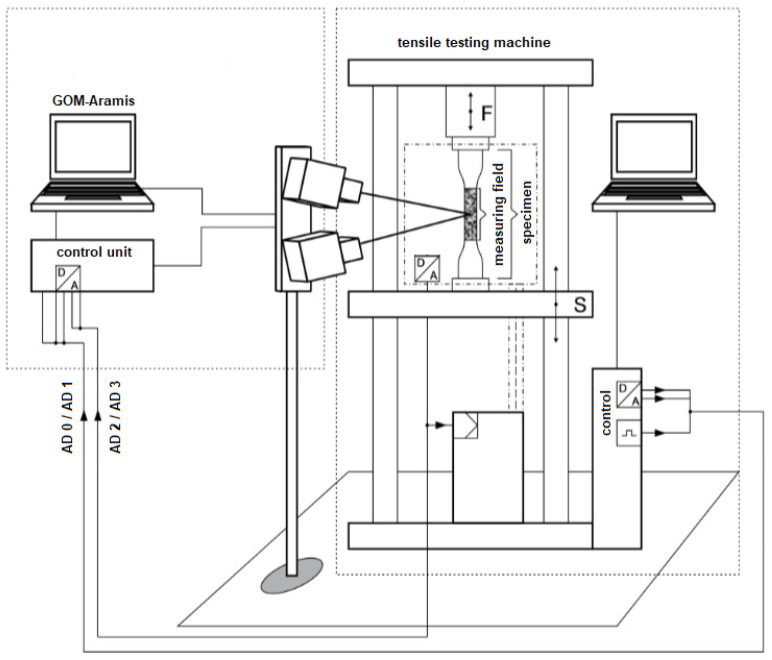
Schematic structure of the GOM measuring system [28].

The tensile tests are carried out using GOM’s Aramis^®^ optical measuring system [23]. The system works on the principle of digital image correlation. Two high speed cameras are used to record changes on the specimen surface. A special pattern, also known as a stochastic pattern, is applied to the tested specimens. The gray value distribution on the surface enables the system to assign coordinates to the individual image pixels. The first image of the test was used as a reference image and is divided into many small rectangular areas. As the specimen deforms, further images are taken as a result of changes in force or length, allowing comparisons to be made. The Aramis^®^ software calculates new coordinates based on the displacements and deformations of the individual image points. From the available displacements of the individual points, the strain and deformation quantities could be determined [2]. The schematic structure of the GOM measuring system is shown in Figure 3. The test conditions are in accordance with DIN EN ISO 527-3 [22], where a quasi-static tensile load is used to test the specimens to failure. The test speed is 1 mm/min, which is kept constant throughout the entire load range. In order to reduce the testing time, the speed could be increased up to 10 mm/min after the determination of the Young’s modulus from a nominal strain of 0.5% according to the standard [22]. This is deliberately not done, because polymers exhibit a time-dependent material behavior (visco-plastic behavior) which directly affects the stress-strain curves that are required for the FEM simulations of different infill strand orientations.

## 5. Results of Tensile Test

**Figure 4:**
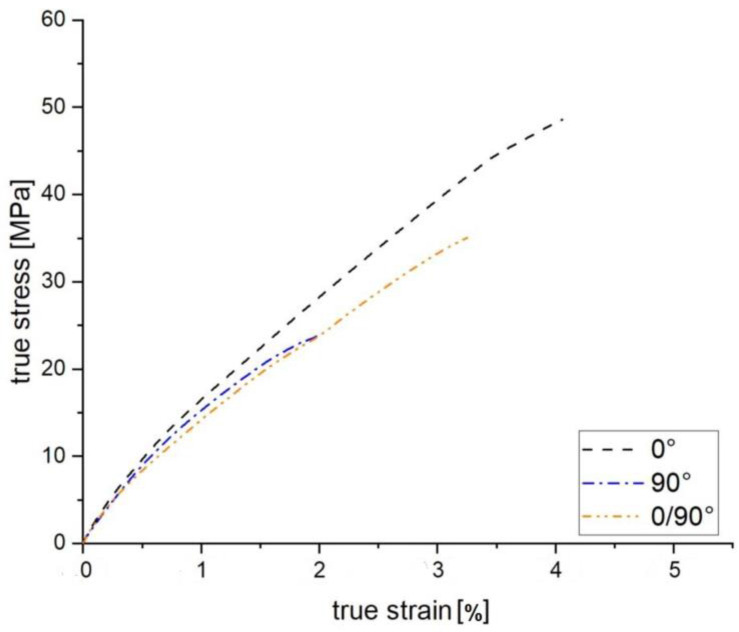
Tensile test results of FFF manufactured specimens with different infill orientations of 0°, 90° and 0°/90°.

The tensile test results show a clear influence of the strand orientation on the mechanical properties of the FFF produced parts (see Figure 4). Here, the average of the stress-strain curves of 15 tensile test results for each orientation are shown. As can be seen from the results, test specimens with an 0° infill strand orientation evoke maximum stress values during tensile loading. At approx. 50 MPa, the maximum stress at failure is more than double that of the specimens tested with an infill orientation of 90°. For the structural design of additive components, it is recommended that the direction of compression of the strands is in the direction of loading to maximize the strength of the component. However, this only applies to uniaxial tensile loading.

Based on the results of the 90° specimens, the influence of the strand orientation becomes obvious. In this variant, failure of the component occurs earliest. The reason for this is the boundary surfaces of the individual strands. These are parallel to the tensile stress, which means that an enormous stress concentration can form between the strand joints. Accordingly, the specimens fail at this point. This failure behavior of the additive components is discussed in more detail in Section 7.1 on crack development in FFF components. Compared to the test specimens with infill strand orientation of 0/90° in the flexural test, the modification of the specimens for the tensile test results in an increase of the mechanical properties. The alternating strand structure of 0° and 90° thus represents an optimization of the 0° specimen. The strands at 0° to the loading direction can compensate for the weak points in a tensile load and lead to an improvement in the mechanical properties.

**Figure 5:**
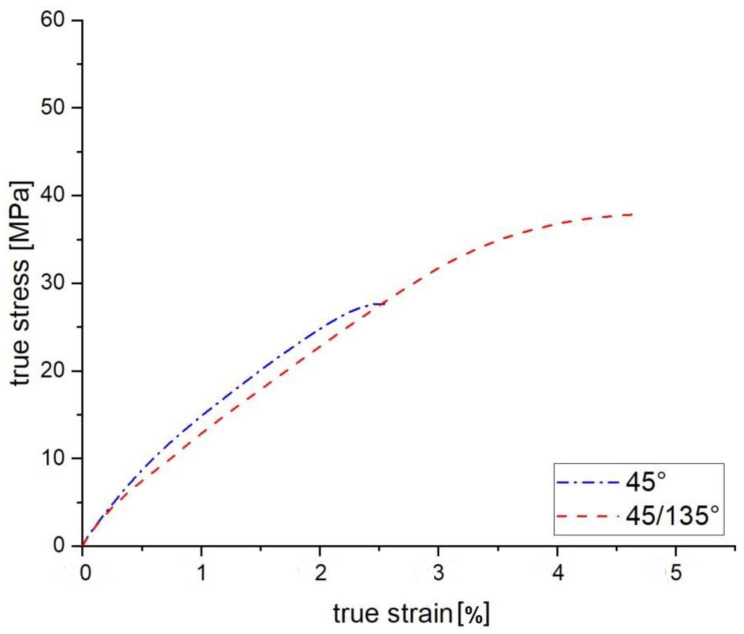
Tensile test results of FFF manufactured specimens with 45°and 45°/135° infill orientation.

The 45° and 45/135° tensile specimens show a similar basic structure. However, under uniaxial loading the unidirectional structure proves to be the worse variant. In contrast, the alternating structure has significantly higher ultimate tensile strength in a range of 30–40 MP as well as strain at failure of 2.5–5% as can be seen in Figure 5. The discrepancy in the characteristic values is due to the different position of the interfaces.

This is due to the alternating structure, which means that the crack cannot grow along the weak point. Instead, crack propagation through the individual layers is observed, which has a negative effect on the deformation behavior. It can be shown that the quasi-static mechanical properties are in approximate agreement with the values available in the literature, both in terms of directional dependence and the quasi-static mechanical behavior [29,30].

## 6. Optical Measurement and FEM Simulation

The optical measuring system from GOM is a non-contact system based on the principle of digital image correlation. As a result of the shift in the stochastic pattern, the system is able to determine the necessary characteristic values of Young’s modulus and transverse contraction coefficient for the FEM simulation. In addition to determining the characteristic values, the optical system provided a comparison with the real measured data for numerical simulation methods. By deforming the surface of the tensile specimen as a result of changes in force or length, the samples could be analyzed with the Aramis^®^ software after the tensile test. In this case, a numerical study of a defined area of the tensile specimen surface is carried out using the measurement data obtained. In this case, the central area of the tensile specimen is examined in more detail, as the greatest changes in deformation and elongation are expected here. The results of the measurement system are then compared with the corresponding calculations from the FEM simulation.

### Modeling in FEM Software

The CAD-geometry as well as the reconstructed tensile test specimens are to be simulated using the Abaqus^®^ FEM software in order to predict the mechanical behavior for additive manufactured components. Before a finite element simulation can be started, modeling must be carried out. This includes mesh discretization and the definition of the boundary conditions.

**Figure 6:**
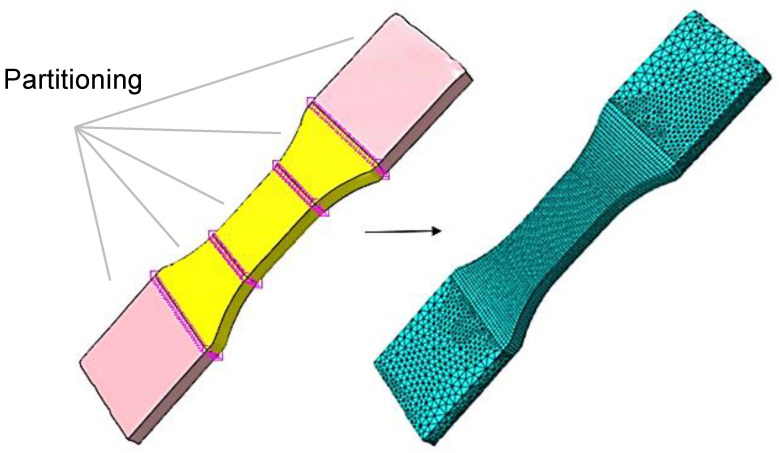
Generated FEM mesh of a reconstructed geometry.

In the model pre-processing step, the tensile specimen must be partitioned into finite elements. In order to achieve a high accuracy of the simulation result, quadratic hexahedral elements are used for the central area of the tensile specimen (compare Figure 2) [31]. In comparison to a tetrahedron mesh, this mesh has more integration points within one element. The shoulder regions of the tensile test specimen are meshed with quadratic tetrahedron elements, as these are located in the clamping region of the tensile specimen and therefore do not provide any significant information on mechanical behavior. To enable the use of various mesh types, the testing and clamping areas of the tensile specimen have to be subdivided. A defined number of elements can then be assigned to these areas (see Figure 6).

The material behavior is assumed to be isotropic elastoplastic. For each direction, the Poisson’s ratio, the Young’s modulus and true stress and plastic strain curves are obtained from the test results. The von Mises yield criterion (J2–plasticity) *F* is used to describe the plastic deformation. Accordingly, yielding occurs when the von Mises equivalent stress *σ_eq,vM_* has the same value as a predefined function *σ_F_*. The deformation behavior of a tensile-loaded polymer can thus be well described [32].

(1) $$F=\sigma_{eq,vM}-\sigma_{F}\sqrt{\frac{1}{2}\left[{\left(\sigma_{11}-\sigma_{22}\right)}^{2}+{\left(\sigma_{22}-\sigma_{33}\right)}^{2}+{\left(\sigma_{33}-\sigma_{11}\right)}^{2}\right]+3\left(\sigma_{12}^{2}+\sigma_{23}^{2}+\sigma_{31}^{2}\right)}-\sigma_{F}=0$$

Considering hardening, the equation for the flow condition *F* is as follows.

(2) $$F=\sigma_{eq,vM}-\sigma_{F}-R\left(\varepsilon^{p}\right)$$

The variable *R*(*ε^p^*) represents a function that can be used to describe strain hardening as a function of the plastic strain *ε^p^*. In semi-crystalline polymer materials, an increase in stress in the form of strain hardening is observed mainly in the plastic region.

The von Mises criterion is also used to describe semi-crystalline polymer materials in combination with the strength assumption by considering equivalent stress (shape change hypothesis) [31].

(3) $$A_{vM}<\frac{\sigma_{eq,vM}}{\sigma_{max}}{\;}_{\;}$$

The utilization rate $A_{vM}$ corresponds to the ratio of the local stress in the FEM simulation $\sigma_{eq,vM}$ to the tensile strength $\sigma_{max}$. In addition to the von Mises failure criterion, the Rankine criterion is considered based on the following equation:

(4) $$A_{NH}<\frac{\sigma_{eq,NH}}{\sigma_{max}};\;\sigma_{eq,NH}=\max\left|\sigma_{1},\sigma_{2},\;\sigma_{3}\right|$$

Similarly, the maximum logarithmic strain (LE) is investigated as the third failure criterion.

(5) $$A_{LE}<\frac{\epsilon_{LE}}{\varepsilon_{max}};\;\epsilon_{LE}=\max\left|\varepsilon_{1},\varepsilon_{2},\;\varepsilon_{3}\right|\;$$

In Rankine’s criterion, the maximum normal stress is used as the limit. The von Mises criterion, on the other hand, uses the combination of normal and shear stresses as the limit value and also considers the energy stored in the material. In addition, the maximum strain criterion is also investigated as it can describe failure in much more detail, particularly in the case of ductile materials. This is due to the convex stress-strain behavior of the PLA material under investigation. All three criteria are validated by comparing the fracture stresses from the test results with respect to different infill strand orientations.

## 7. Comparison of Reconstructed Geometry and CAD Geometry

**Figure 7:**
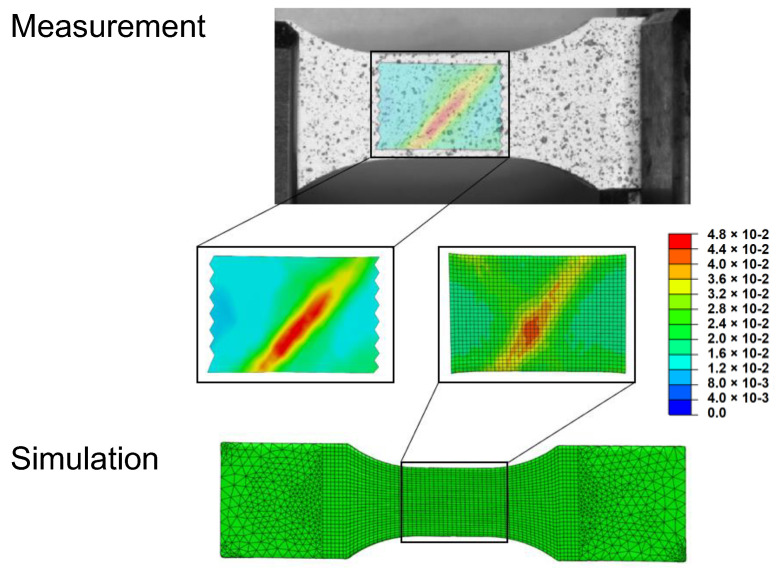
Comparison of the FEM–simulation and test results using GOM Aramis^®^ by means of highly stressed areas (true strains).

To illustrate the advantage of the optical measurement and the possible comparison with the simulation, the results are analyzed in terms of strains on the surface of a 45° strand orientated specimen. In this evaluation, the influence of the strand orientation on the strain behavior of the FFF component became very clear. This is due to the local strain that forms in the direction of the individual extruded strand. From Figure 7, it can be seen that the fracture of the FFF component occurs in the strand direction at the position of the highest strain concentration. This dependence is also evident from the FEM simulation results. The local strain results in Aramis^®^ and Abaqus^®^ showed a similar formation in the present case. Thus, with the quasi-static implicit FEM simulation carried out, an accurate prediction of the resulting local strains could be made.

**Figure 8:**
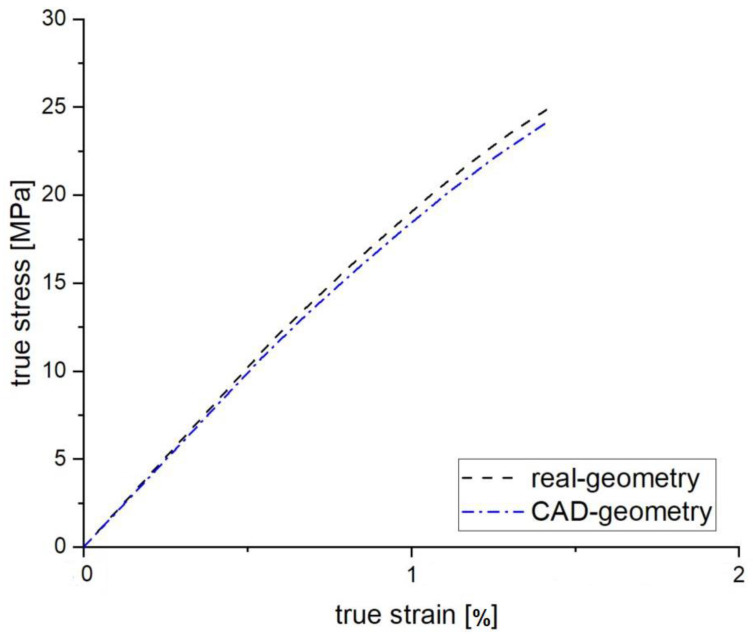
Comparison of the FEM-simulation results from tensile tests of real geometry and CAD geometry for an 0°/90° infill orientation.

Using the reverse engineered tensile specimen and the associated CAD model, a comparison is made with the two solid models using FEM simulation for an infill orientation of 0°/90°, which is expected to be the common strand orientation in real applications. The focus here is on investigating the geometric influence. Due to shrinkage, the reconstructed tensile specimen geometry differs significantly from the CAD model. For the simulation, the same characteristic values from the tensile test are assigned to both model variants. Furthermore, the manipulation or discretization of the models in the FEM software is performed with the same settings. The two curves show a similar behavior (see Figure 8). However, minor discrepancies can be observed which are due to the geometry deviation of the reverse engineered tensile specimen. This is particularly evident in the strength of the component.

Another finding is that the same input parameters and boundary conditions for both simulated tensile specimen variants lead to different simulation results. Traceability of the geometry is therefore essential for accurate prediction of mechanical properties.

Likewise, the geometry deviation has an effect on the deformation behavior and thus on the stress and strain as well as on the Young’s modulus. The values are compared in Table 2. In particular, the stiffness and the stress at break (tensile strength) differ significantly. The averaged Young’s modulus for this tested orientation is 1960 MPa.

### 7.1. Simulated Deformation Behavior and Failure

**Figure 9:**
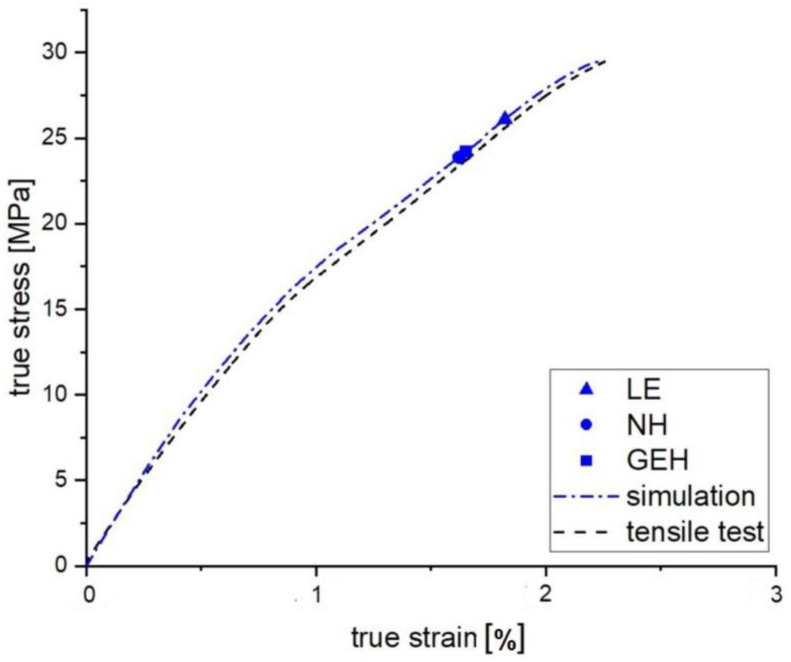
Stress-Strain Curve: Comparison of FEM simulation and experimental tensile test of FFF manufactured specimens with infill strand orientation of 45°.

Using the FEM software, a comparison is made with the mechanical tensile test results in order to verify whether a numerical description for the approximate solution of the FFF manufactured tensile specimens would be feasible. Figure 9 shows the experimental and simulated tensile test results of the FFF manufactured specimen with an infill strand orientation of 45°. It can be seen that the stress-strain curves are almost identical. It can therefore be concluded that an approximate prediction of the quasi-static FEM calculation is possible using the model presented.

The three criteria examined reach their failure criterion at different stress states. This is due to the weighting of each stress state. Table 3 shows the failure stresses of the criteria. By determining the failure stress from the experimental tensile tests, a comparison can be made with the failure criteria for the maximum utilization of the material or strand orientation potential.

With respect to the uniaxial loading direction of the test, the two stress-based criteria show only minor differences in failure stress. Apart from this failure criterion, the failure can be described using the maximum logarithmic strain (LE). For the 45° tensile specimen, this description provides the most reliable prediction of the mechanical behavior of the additive tensile specimen. This is evidenced by the smaller deviation of the logarithmic strain from the elongation at break. In contrast to the normal stress hypothesis, the von Mises hypothesis gives a better approximation of the fracture stress.

Due to the large variance in elongation at break between the test result and the failure value according to the Rankine failure criterion (NH), a realistic prediction of mechanical properties using this parameter is not recommended. This indicates that maximum utilization of the material potential is not possible.

The comparison between the simulation and the tensile test results for the remaining infill strand orientations are summarized in Table 4. It can be seen that with the maximum logarithmic strain (true strain, LE), the simulation results can be best predicted with this variable, especially for the 0°/90° cross laminated direction. Thus, for all strand orientations considered, failure can be best predicted using the strain-based criterion.

There is currently no known study in the literature that can be used to compare the deviations of the considered failure criteria and the associated evaluation of the strength of FFF 3D-printed components made of PLA.

## 8. Conclusions

This work investigates the numerical and experimental analysis of the mechanical properties of additively manufactured components using the FFF method, considering manufacturing induced geometric deviations. For the experimental work, tensile specimens are 3D printed from PLA material with different infill strand orientation (0°, 45° and 90°) and their corresponding mechanical properties (true stress and true strain) are determined.

To make a realistic prediction of the deformation behavior and strength of the tensile specimens made of PLA, the real geometry is scanned using Computer Tomography (CT scan). The focus here is on investigating the geometric influence. Due to shrinkage, the reconstructed tensile specimen geometry differs significantly from the CAD model.

Tensile tests of the investigated specimens showed that the orientations have a strong influence on the deformation behavior and the strength of the 3D printed PLA material. The effect on the mechanical behavior is particularly evident for an orientation of 0° and 90° to the loading direction. When the orientation is transverse to the tensile loading, the FFF additive manufactured tensile specimen fails relatively early. On the other hand, an orientation perpendicular to the tensile stress indicates maximum stress values. Stress at failure varies from 22.5 N/mm^2^ (90° orientation) to 51 N/mm^2^ (0° orientation) and the strain at failure varies from 2.2% (90° orientation) to 4.3% (0° orientation). Higher values for both stress and strain can be achieved using the 0° orientation.

Furthermore, the tensile test results are compared to a quasi-static FEM analysis of the tensile specimen CAD geometry. In addition, the FEM simulations are repeated for the actual 3D printed tensile specimen geometries reconstructed using CT scanning. It can be shown that by reconstructing the actual geometry, it is possible to accurately predict the behavior of FFF additive manufactured components under mechanical loading. The difference between the simulated and actual Youngs’s modulus is only about 3.5%. The difference with the ideal CAD geometry is greater (7%). Plastic deformation behavior and damage can also be predicted more accurately. The simulated stress and strain at failure differ slightly from the measured results.

The comparison of the real geometry and the CAD geometry by FEM simulation showed that the mechanical behavior depends on the simulated geometry. Shrinkage was found to have a significant influence on the prediction of mechanical properties.

As a result of using the optical measurement system and the FEM simulation, a comparison is made of the local strain that occurs during the tensile test. It was found that the strain propagates along the strand orientation until the component fails. This strain concentration is also represented in the simulation, allowing the resulting local strains to be predicted by numerical calculation.

Based on the FEM simulation results, it is found that a prediction of the mechanical properties of an additively manufactured component can be well described. In addition, the logarithmic strain provides a realistic prediction of failure. This value can also be used to assess the strength of complex geometries. With reference to the commonly used cross-layer orientation in the FFF process, it is recommended to use the material properties of the 0–90° orientation as an input variable to the FEM simulation.

Future research works could look at how the local strand orientation can be considered in the FEM-simulation. The question to be answered is how the mechanical properties can be coupled with the strand orientation and how the resulting anisotropy can be coupled locally at each node in the FEM model. This opens up the possibility of using the material properties determined in the course of this work as a basis for fitting elastoplastic, anisotropic material models in the FEM. For example, Hill’s plasticity could then be used to describe directional dependencies in plastic deformation. In addition to the tests carried out, shear tests with different directions of deflection should be carried out in order to couple the change in shape with the directional dependencies.

## Figures and Tables

**Table 1:** 3D Printing process parameters of the tensile specimens.

Parameter	Value
Extrusion temperature	220 °C
Platform temperature	70 °C
Layer height	0.1 mm
Processing speed	60 mm/s
Nozzle diameter	0.2 mm
Outline lines number	2
Infill percentage	100

**Table 2:** Stress based comparison of real-geometry and CAD-geometry for an infill orientation of 0°/90°.

	True Stress [MPa]	True Strain [%]	Young’s Modulus [MPa]
Simulation Results			
**real-geometry**	29.51	2.26	**2031**
**CAD-geometry**	26.1	1.82	**2097**
**Test results**			**1960**

**Table 3:** Variance of FEM simulation and experimental tests results for an infill orientation of 45°, by focusing on different failure criteria.

	True Stress [MPa]	Variance	True Strain [%]	Variance
**tensile test**	29.51	**-**	2.26	**-**
**log. strain (LE)**	26.1	**11.56%**	1.82	**19.47%**
**Rankine**	23.87	19.11%	1.62	28.32%
**von Mises**	24.18	18.06%	1.65	26.99%

**Table 4:** Variance of FEM simulation and experimental tensile test results of FFF manufactured specimens for 0°-, 90, 0°/90° and 45°/135° infill orientations, with reference to different failure criteria and the maximum stress and maximum strain.

Specimen 0°	True Stress [MPa]	Variance	True Strain [%]	Variance
tensile test	49.09	-	3.57	-
log. strain (LE)	40.92	**16.64%**	2.65	**25.77%**
Rankine	39.94	18.64%	2.56	28.29%
von Mises	40.43	17.64%	2.61	26.89%
**Specimen 90°**	**True Stress [MPa]**	**Variance**	**True Strain [%]**	**Variance**
tensile test	23.72	-	1.97	-
log. strain (LE)	20.26	**14.59%**	1.56	**20.81%**
Rankine	18.47	22.13%	1.39	29.44%
von Mises	18.78	20.83%	1.42	27.92%
**Specimen 0°/90°**	**True Stress [MPa]**	**Variance**	**True Strain [%]**	**Variance**
tensile test	35.8	-	3.29	-
log. strain (LE)	34.64	**3.24%**	2.69	**18.24%**
Rankine	31.09	13.16%	2.24	31.91%
von Mises	33.57	6.23%	2.53	23.10%
**Specimen 45°/135°**	**True Stress [MPa]**	**Variance**	**True Strain [%]**	**Variance**
tensile test	34.22	-	3.74	-
log. strain (LE)	32.53	**4.94%**	3.33	**10.96%**
Rankine	28.99	15.28%	2.82	24.60%
von Mises	29.37	14.17%	2.87	23.26%

## Data Availability

Not applicable.
